# Newman's Fortreviver

**Published:** 1919-07-05

**Authors:** 


### Newman's Fortreviver.

A LIQUEUR TONIC.

(41-2 Upper Rathbone Place, London, W. 1.)

We published a notice of Newman's Fortreviver in The Hospital of April 27, 1918, page 86, in the course of which we expressed the opinion that as a domestic tonic, appetiser, or refreshing drink within the limitations of such preparations, it is to be recommended, and is likely to obtain a considerable sphere of usefulness. Since that opinion appeared Fortreviver has secured very wide publicity, and it can now be readily obtained at most of the large stores and elsewhere. Its medicinal properties depend to some extent upon its gastronomic ones, and also upon its actual constituents, the most important of these latter being those contained in fruit juices and vegetable-iron compounds. Its taste is pleasant, slightly bitter and slightly astringent. It is a vegetable tonic which has the appearance, and to some extent the taste, of wine, but is non-alcoholic. It may be taken as a tonic in doses of about a port-wine glassful three times a day between meals, or diluted with soda-water or milk to taste. It may also be taken with wine, with whisky, or with brandy-and-soda. It is further claimed that it improves the taste of hock or claret cup. The demand for it seems to be considerably on the increase, and this vegetable tonic has evidently come to stay. Bitter and slightly astringent tonics are in great favour with some people, and those of our readers who are in search of a change might find the taste of Fortreviver pleasant and settle down to its use with advantage and satisfaction.

